# The epidemiology of laryngeal cancer in Brazil

**DOI:** 10.1590/S1516-31802004000500002

**Published:** 2004-09-01

**Authors:** Victor Wünsch

**Keywords:** Laryngeal cancer, Epidemiology, Incidence, Mortality, Risk factors, Neoplasias laríngeas, Epidemiologia, Incidência, Mortalidade, Fatores de risco

## Abstract

The city of São Paulo exhibits one of the highest incidences of laryngeal cancer in world and Brazil presents remarkable occurrence, compared with other Latin American countries. Around 8,000 new cases and 3,000 deaths by laryngeal cancer occur annually in the Brazilian population. In the city of São Paulo, incidence rates for laryngeal cancer among males have been decreasing since the late 1980s while, among females, the rates have shown a stable trend. This phenomenon is probably the expression of changes in gender behavior related to tobacco smoking. Several risk factors are involved in the genesis of laryngeal cancer. The most important are tobacco smoking and alcohol intake, but occupational hazards have also been associated with the disease, such as asbestos, strong inorganic acids, cement dust and free crystalline silica. Additionally, salted meat and total fat intake have been linked to elevated risk of laryngeal cancer. Conversely, several studies have confirmed that fruits, raw leaf vegetables and legumes protect against this cancer. Some researchers have postulated a possible association between laryngeal squamous cell carcinoma and human papilloma virus (HPV), but this is not universally accepted. Gastroesophageal reflux disease is weakly, but consistently correlated with laryngeal cancer. Familial cancer clusters, particularly of head and neck tumors, seem to increase the risk of laryngeal cancer. Some genetic polymorphisms, such as of genes that code for xenobiotic-metabolizing enzymes, have shown elevated risk for laryngeal cancer according to recent studies. Public health policies regarding the control of tobacco smoking and alcohol consumption, and also surveillance of carcinogen exposure in occupational settings, could have an impact on laryngeal cancer. No proposals for screening have been recommended for laryngeal cancer, but one diagnostic goal should be to avoid treatment delay when suspected symptoms have been observed.

## RESUMO

A cidade de São Paulo apresenta uma das mais altas incidências de câncer de laringe no mundo e o Brasil uma ocorrência expressiva quando comparada com outros países da América Latina. Em torno de 8.000 casos novos e 3.000 mortes pela doença atingem anualmente a população brasileira. Na cidade de São Paulo, as taxas de incidência por câncer de laringe mostram-se decrescentes desde o final da década de 1980, porém, entre as mulheres as taxas revelam-se estáveis. Estas tendências, possivelmente, expressam as mudanças no comportamento de homens e mulheres em relação ao consumo de cigarros. Vários fatores de risco são relacionados ao câncer de laringe. Os mais importantes são tabagismo e consumo bebidas alcoólicas, porém exposições a cancerígenos ocupacionais também são associadas à doença, como o amianto, os ácidos inorgânicos fortes, a poeira de cimento e a sílica cristalina livre. O consumo de carne salgada e gorduras está relacionado com excesso de risco de câncer de laringe. Por outro lado, vários estudos têm confirmado que frutas, vegetais crus e legumes protegem contra este câncer. Há referências da possível associação entre carcinoma espinocelular de laringe e o papiloma vírus humano (HPV), mas este fato não está comprovado. O refluxo gastro-intestinal relaciona-se de forma tênue, porém consistente com o câncer de laringe. Aglomeração de câncer na família, particularmente de tumores de cabeça e pescoço, aumenta o risco de câncer da laringe. Polimorfismos genéticos, especialmente de genes envolvidos na ação de enzimas responsáveis pela metabolização de cancerígenos, apresentam associação com o câncer de laringe de acordo com estudos recentes. Políticas de controle do tabagismo e consumo excessivo de álcool, bem como a vigilância de exposições a cancerígenos ocupacionais, terão benéficas repercussões no câncer de laringe. Não há recomendação para o rastreamento de câncer da laringe, todavia uma vez realizado o diagnóstico o tratamento médico imediato influenciará na sobrevida do paciente.

## INTRODUCTION

Laryngeal cancer is generally uncommon in males and very rare in females. Laryngeal tumors represent around 2.0% of all cancers in Brazil, corresponding to approximately 8,000 new cases annually.^1^ They account for 3.8% in men and 0.6% in women of all deaths by cancer, corresponding to around 3,000 deaths annually.^2^ The disease is predominantly found in patients aged from 50 to 70 years, although in developing countries many cases are diagnosed in individuals in their fifth decade of life. In a case-control study conducted in the Metropolitan Region of São Paulo, between January 1999 and December 2001, 63% of the laryngeal cancer cases occurred in the age group from 50 to 70 years.^3^ The lifetime risk (30-74 years) of laryngeal cancer among males has been found to be less than 1% in most countries for the generation born in 1915. But in São Paulo the lifetime risk for the 1915 cohort was found to be greater than 1%.^4^ Cancer at this anatomical site was rarely diagnosed until about 1860, when Garcia developed the laryngoscope.^5^ Although the larynx may be the site of primary neoplasms such as sarcomas, adenocarcinomas, cylindromas, lymphomas or histiocytomas, these are rare. Laryngeal cancer relates almost exclusively to squamous cell carcinoma of varying degrees of histological differentiation. In the case-control study previously mentioned,^3^ out of a total of 129 cases of laryngeal cancer screened in seven hospitals in the city of São Paulo, 121 (93.7%) were classified as squamous cell carcinoma. Another 0.8% was classified as nonspecific “carcinoma”, 0.8% as verrucous carcinoma, 0.8% as tubular adenocarcinoma and 0.8% as neuroendocrine carcinoma. The remaining four cases (3.1%) had tumors associated with both the larynx and the pharynx (data not published). In this review the term laryngeal cancer relates to squamous cell carcinoma.

The larynx is divided into three anatomical regions: supraglottic, glottic and subglottic larynx. The glottis consists of the true vocal cords and the mucosa of the anterior and posterior commissures. Cancers of the supraglottic or subglottic areas have a high tendency to involve adjacent organs, such as the hypopharynx by infiltration and the cervical lymph nodes by lymphatic spread. The descriptive epidemiology of laryngeal cancer is complicated by the difficulty in clinically distinguishing between tumors of the supraglottis or epilarynx and those of the hypopharynx, since the larynx has a complex anatomy that is closely related to the hypopharynx, and also by the international variation in the proportions of cancers of the glottis or endolarynx (including vocal cords) among all laryngeal cancers.^4^ Most failures in the treatment of laryngeal cancer are due to the difficulty in eradicating the locoregional disease, and a large number of such laryngeal cancer patients die from the disease.^5^

### Incidence patterns

Worldwide estimates for 2005 predict more than 160,000 new cases of laryngeal cancer in males and 22,000 cases among females.^6^ These predictions only account for 1.7% of all new cancer cases around the world. The male/female ratio (almost 7:1) is higher than for cancer at any other site, thus emphasizing the rarity of laryngeal cancer among females.^7^ This international male/female ratio for the incidence of laryngeal cancer has been based particularly on data from developed countries. A similar male/female ratio (almost 6:1) was observed among cases in the case-control study conducted in the Metropolitan Region of São Paulo from 1999 to 2001.^3^ Among men, the highest incidence rate during the early 1990s was reported from Zaragoza, Spain (17.1 cases per 100,000 males per year). The areas with high incidence for men (> 10/100,000 per year) are France, northern Italy, Spain and Portugal, various areas of central Europe, southern Brazil, Uruguay and western Asia (Table 1).^8-11^ In western Asia, laryngeal cancer accounts for more than 6% of all cancers among men. Areas with low incidence include most regions of Africa and eastern Asia for which cancer incidence data is available, Andean countries in South America and areas of Central America, Australia and New Zealand, Canada except Quebec and several but not all countries of northern Europe. The lowest rate has been reported from Qidong, China (0.7 per 100,000 per year). In Brazil, the city of Goiânia showed an adjusted rate of 6.4 cases per 100,000 males for the period from 1995 to 1998^12^ and São Paulo 14.9 per 100,000 males in 1998.^10^

**Table 1:** Laryngeal cancer incidence among males. Selected countries (whole territory or specific places), circa 1990-1995^8-11^

Annual age-adjusted rate (world standard population) per 100,000		
< 5	5-10	> 10
Cali, Colombia	Bombay, India	Ahmedabad, India
Costa Rica	Canada	Vas County, Hungary
Gambia	**Goiânia, Brazil**	Cuba
Osaka, Japan	Hong Kong, China	Isère, France
Quito, Ecuador	Manila, Philippines	**São Paulo, Brazil**
Setif, Algeria	Puerto Rico	Turin, Italy
Qidong City, China	Saarland, Germany	USA (among blacks)
Sweden	St. Petersburg, Russia	Vila Nova de Gaia, Portugal
Trujillo, Peru	United Kingdom	Warsaw City, Poland
Western Australia	USA (among whites)	Zaragoza, Spain

Table 2 shows the incidence rates for laryngeal cancer among males, from population-based cancer registers in Brazilian cities. It is difficult to recognize any pattern according to the regions of the country from these data. But it can be stated that, in the majority of these cities, the male incidence is greater than 5.0 per 100,000.^13^ Among women, blacks from the United States have the highest reported incidence. For example, black women in Detroit show an adjusted standardized rate of 2.9/100,000. Other female populations with high incidence include: whites from the United States and in areas of South America, Asia and Europe. The low rates found among women from Spain and France are remarkable.^12^ In Brazil, the rates among females in Goiânia and São Paulo were, respectively, 1.5 and 1.8 per 100,000.^10,12^

**Table 2:** Laryngeal cancer incidence among males. Selected Brazilian cities, circa 1995-2000^1^

Annual age-adjusted rate (world standard population) per 100,000		
< 5	5-10	> 10
Belém, PA	Campinas, SP	Belo Horizonte, MG
Palmas, TO	Goiânia, GO	Cuiabá, MG
	João Pessoa, PB	Federal District
	Natal, RN	Fortaleza, CE
	Recife, PE	Manaus, AM
	Salvador, BA	Porto Alegre, RS
	Vitória, ES	São Paulo, SP

*Brazilian States: AM = Amazonas; CE = Ceará; ES = Espírito Santo; GO = Goiás; MG = Minas Gerais; PA = Pará; PB = Paraíba; PE = Pernambuco; RN = Rio Grande do Norte; RS = Rio Grande do Sul; SP = São Paulo; TO = Tocantins.*

### Mortality patterns

The International Agency for Research on Cancer (IARC) has estimated that there will be around 89,000 deaths due to this cancer among males and 12,000 among females worldwide in 2005.^6^ Mortality from this cancer is particularity high among males from eastern and southern Europe (rates around 6.5 to 7.5 per 100,000). At a second level, South America (southern Brazil, Uruguay and Argentina) shows the highest mortality rates. Mortality due to this cancer is very rare among women, accounting for only 0.4% of all deaths due to cancer around the world.^14^ In Brazil, the highest mortality rates for males (6.2 per 100,000) and females (0.6 per 100,000) have been registered in São Paulo. The lowest rates have been detected in the state of Maranhão for males (0.5 per 100,000) and in the states of Acre and Amapá for females (less than 0.01 per 100,000).^2^

### Incidence and mortality time trends

**Figure 1:**
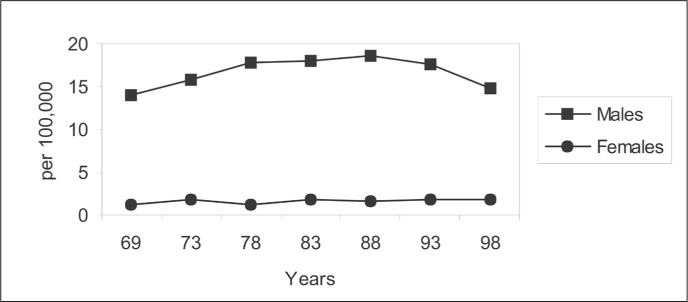
Laryngeal cancer incidence trends among males and females in the city of São Paulo, 1969-1998.^10,16^

The time trends for laryngeal cancer incidence and mortality are consistent with the trends for other cancers associated with tobacco and alcohol. Increasing trends are seen in central and eastern Europe and in most developing countries, while in North America and western Europe the incidence and mortality have either leveled off or are decreasing (Coleman et al. 1993).^4^ But among females, increasing incidence has been reported from Canada, Italy, Denmark, United States and Australia.^15^ In Latin America countries, different incidence trend patterns are suggested for Cuba (steady increase), Puerto Rico (stable) and Cali, Colombia (decreasing).^4^ Figure 1 shows the incidence rate trends for laryngeal cancer in the city of São Paulo. These have declined for males since the late 1980s, but have been stable for females.^10,16^

The mortality trends in Latin America have shown a steady increase for Costa Rica, a small increase for Uruguay and a decrease in young birth cohorts for Chile and Venezuela.^4^ In Brazil, the mortality rates among males went from 2.5 per 100,000 individuals in 1979 to 3.0 per 100,000 in 1999. Among females, the mortality rate (around 0.4 per 100,000) was stable over that same period.^2^ In São Paulo, the mortality rates were stable from 1969 to 1998 for both men and women, with rates ranging from less than 1.0 per 100,000 for females to 6.0 to 8.0 per 100,000 for males.^10^

### Survival patterns

Even though large percentages of patients die from the disease, laryngeal carcinoma is a highly curable disease. In the late 1960s, there was a significant improvement in the five-year survival of patients with laryngeal neoplasms, mostly as a result of improvements in the prognosis of patients with regional disease. According to data from developed countries, the prognosis for all patients with laryngeal cancer has remained unchanged since the mid-1970s, with a relative survival rate of 60-65% after five years, for all stages and all forms of treatment.^5^ But in fact, overall improvement in the prognosis was seen in Europe from 1978 to 1989, where the five-year survival increased from 58% to 63%.^17-19^ Advancing age, regional and metastatic disease (advanced stages of the diagnosed tumor) and also supraglottic cancers have been associated with significantly reduced survival.^5,20,21^ Glottic tumors have a more favorable prognosis than for supraglottic and subglottic tumors, which show poor survival that is similar to the rate seen for cancer of the pharynx.^22,23^ Cancers of the hypopharynx may be attributed to the larynx on the death certificate, and thus not only the survival data but also the mortality data from laryngeal cancer are difficult to interpret.^4^ Two reasons are indicated for the higher rate of control for glottis tumors. First, since cancers of the true vocal cords produce persistent and early change in voice quality, this symptom improves early diagnosis. Second, vocal cord carcinomas tend to be metastatically inefficient, since the vocal cord is very poor in vessels (both lymphatic and blood vessels) for stimulating the angiogenic cascade, thus resulting in a low rate of metastatic spread, occurring in less than 6% of such cases.^21^ Considering the minimal superficial nature of tumors that are limited to the glottis, some authors have recommended that these tumors be treated as carcinoma in situ and not as invasive carcinoma.^24^ Robbins^2^5 conducted a study of disease-free survival among males and females with laryngeal cancer and did not find any significant survival differences between men and women with regard to local and regional tumor control.

Survival rates are lower in developing countries than in developed countries. In a study in India, the five-year relative survival rates were 58.3% for glottic laryngeal cancer cases and 31.4% for supraglottic laryngeal tumor cases. The five-year observed survival was 53.1% for those with localized cancer and 17.8% for patients with regional extension.^26^ Liu et al.^27^ studied fifty-nine laryngeal or hypopharyngeal squamous cell tumors in patients who underwent surgery and found, in a multivariate analysis, that lymph node metastasis was the only statistically significant factor associated with poor prognosis. They also found that expression of proliferating cell nuclear antigen (PCNA), a DNA polymerase accessory protein, and Ki67, a large molecular weight protein whose expression is restricted to the G1 through M phases of the cell cycle and whose function remains unknown, were positively associated with poorly differentiated laryngeal carcinoma and laryngeal carcinoma that was associated with lymph node metastasis. However, these results do not match those obtained in other studies for these biomarkers.^28-30^

### Screening and case searches

Screening is not recommended for laryngeal tumors, since no whole population screening program for laryngeal cancer has been evaluated.^31,32^ Even though preclinical case searches cannot be recommended, one goal should be to avoid medical diagnostic delay when suspected symptoms have been noted.

There is a high incidence of second primary cancers among patients with laryngeal tumors, particularly other head and neck, esophageal or bronchial tumors. However, screening employed after the first treatment has been unsuccessful in yielding significant reduction in mortality from such second primary tumors.^15^

### Risk factors

Squamous cell carcinoma of the larynx is a multifactorial disease influenced by environmental and lifestyle-related factors. Heavy tobacco smoking, excessive alcohol consumption and exposure to chemical influences caused by some occupational hazards are known to be involved as etiological factors in laryngeal tumors. In addition, low educational levels, papilloma virus infection and gastroesophageal reflux have been suggested as cofactors for this cancer, according to some epidemiological studies. On the other hand, a diet rich in fruits and vegetables seems to have a protective effect against laryngeal cancer. Inherited polymorphisms of genes that code for carcinogen-metabolizing enzymes, and also genes responsible for cell cycle control and DNA repair systems, may increase the risk of laryngeal cancer, even though a causal mechanism has not yet been thoroughly demonstrated.^5^

### Tobacco and alcohol

Mortality trend studies on laryngeal cancers performed in France, Britain and Australia in the 1970s suggested that these tumors were related to patterns of tobacco and alcohol consumption in different generations of males.^33,34^ Ecological studies linking the risk of laryngeal cancer in different populations to the *per capita* consumption of tobacco and alcohol have generally shown a closer association with the latter factor.^35^ Chronic consumption of tobacco and alcohol independently increases the relative risk of laryngeal cancer in a dose-dependent fashion. The relative risk is generally far higher for smoking than for alcohol consumption. In a case-control study, Wynder et al.^36^ estimated an odds ratio for tobacco consumption in relation to laryngeal tumors of 13.2. These authors noted that the combined effect of tobacco and alcohol seemed to be multiplicative, as confirmed in a reanalysis of those data by Flanders & Rothman.^37^ In subsequent case-control studies,^38,39^ similar conclusions were reached.

In most studies, the risk of laryngeal cancer among alcoholics has been found to be twice as high as among those not dependent on alcohol.^35^ Several studies have confirmed a dose-response relationship, in which the risk of laryngeal cancer increased with increasing daily consumption of alcohol.^40-43^ A case-control study on laryngeal cancer in São Paulo found an adjusted odds ratio of 7.5 for heavy smokers and 3.7 for heavy drinkers.^3^ In another study conducted in southern Brazil from 1986 to 1989, no interaction between tobacco smoking and alcohol consumption was observed in the risk of laryngeal cancer,^44^ clearly negating the interaction found in other studies.^37,39^

### Occupation

Low educational levels and some occupations are associated with high risk of laryngeal cancer. A large proportion of patients with laryngeal cancer are blue-collar workers exposed to a variety of chemical hazards like polycyclic aromatic compounds, cement dust, metal dust, asbestos, varnish and lacquer.^45-47^ Since the 1970s, exposure to asbestos has been treated as a risk factor for laryngeal cancer.^48,49^ In a case-control study, Shettigara and Morgan^50^ found that asbestos was more strongly associated with the disease than were smoking and alcohol. Since then, other studies have noted increased laryngeal cancer among asbestos-exposed workers.^51-54^ In 1992, based on results from several studies, IARC concluded that there was sufficient evidence to classify sulfuric acid and other strong inorganic acid mists as human carcinogens, including the risk of developing laryngeal, lung and nasal sinus cancers.^55^ This elevated risk of laryngeal cancer was confirmed later in a cohort study in the United States among steel industry workers exposed to acid mists.^56^ Studies have shown that construction workers display higher risks of acquiring laryngeal cancer.^45,47^ A population-based case-control study performed in Germany has confirmed that exposure to cement dust is an independent risk factor for laryngeal cancer.^57^ Occupations related to working with wood have been found to affect the risk of laryngeal cancer independently of smoking status in a case-control study from the United States.^36^ Workers in wood-related occupations (wood-workers and furniture workers) exposed for over 20 years showed elevated risk in a case-control study conducted in Madrid.^58^ A multicenter European case-control study found an elevated risk of laryngeal cancer (odds ratio 1.8; 95% confidence interval 1.3–2.7) for males aged 55 years who were exposed to wood dust.^54^ However, other studies that have explored such an association have either indicated that there is no risk or that the estimated risk is low.^59^ In an IARC monograph on the rubber industry,^60^ no relationship between working in that industry and laryngeal cancer was identified. However, a cohort study conducted in São Paulo among rubber industry workers identified four cases of laryngeal cancer. These cases accounted for 6.0% of all cancers identified in the study: three times higher than expected.^61^ Asbestos, vulcanization fumes and, particularly, nitrosamines have been considered as the possible causative agents of cancer in the rubber industry, in addition to naphthylamine.^60^ A case-control study from the United States found risk factors of more than 3.0 for railroad industry workers, sheet metal workers, grinding wheel operators and automobile mechanics.^62^ De Stefani et al.,^53^ in a case-control study conducted in Uruguay, found an elevated risk of laryngeal cancer among butchers, vintners, bakers and car assemblers. In addition, they found that asbestos, mists from strong inorganic acids and pesticide exposure were associated with increased risk of laryngeal cancer. A case-control study performed in São Paulo^3^ found elevated risk among workers exposed to respirable free crystalline silica, soot, fumes in general, and live animals.

### Nutrition and diet

Case-control studies conducted in Uruguay^63-65^ have indicated that dietary patterns with high consumption of salted meat and high total fat intake are associated with laryngeal cancer. Total fat intake combines its effect multiplicatively with tobacco smoking. On the other hand, intake of plant foods, fruits, raw leaf vegetables and legumes are associated with protection against laryngeal cancer. Case-control studies conducted in Europe have also confirmed these relational patterns between nutrition and laryngeal cancer.^66-68^

### Viruses

Studies have postulated an association between laryngeal squamous cell carcinoma and human papilloma virus (HPV), types 16, 18 and 33.^69^ One case-control study identified increased odds ratios for exposure to oncogenic HPV types and laryngeal cancer, as well as laryngeal leukoplakia, but the data were not statistically significant after controlling for tobacco smoking and alcohol consumption.^70^ HPV positivity is much more common in oropharyngeal cancers and it has been postulated that HPV plays a role in a fraction of cancers of the oral cavity and pharynx.^71^ However, an analogous role for HPV in larynx cancer is less clear.

The relationship between HPV infection and laryngeal cancer is taken for granted, since recurrent respiratory papillomatosis is caused by proliferative growth induced by HPV 6 and 11 infection in the laryngeal epithelium.^71^ In a pilot study on a series involving 50 laryngeal cancer cases forming part of a multicenter case-control study (São Paulo, Rio de Janeiro, Goiânia, Porto Alegre, Pelotas and Buenos Aires), the presence of HPV-DNA detected through the polymerase chain reaction (PCR) was identified only in one case (unpublished data).

### Gastroesophageal reflux disease

Over recent years, gastroesophageal reflux as an independent carcinogenic factor and co-carcinogen in association with smoking and alcohol consumption has stirred great interest. Two case series in France and Italy^72,73^ have confirmed that gastroesophageal reflux is often present in patients with squamous cell carcinoma of the pharynx and larynx. One case-control study has identified a small but significant elevation of the risk of laryngeal cancer after controlling for age, sex, tobacco smoking and alcohol consumption.^74^ Weaver^75^ performed a systematic review of studies that explored associations between gastroesophageal reflux and sinusitis, otitis media and laryngeal malignancy. Based on the analysis of 18 articles, he found weak support for a positive association between gastroesophageal reflux disease and laryngeal malignancy. However, the mechanism for carcinogenic action is not yet clearly understood.

### Familial clusters and genetic susceptibility

Genetic susceptibility to environmental risk factors and carcinogens is by and large recognized. In a case-control study conducted in Brazil, an elevated risk of developing squamous cell carcinoma of the head and neck was detected in individuals with a first-degree relative who had had some type of cancer, but the risk was higher (odds ratio 3.65; 95% confidence interval 1.97–6.76) if the relative had had head or neck cancer.^76^ Based on these results only, it is difficult to set apart the influence of heredity versus shared environmental factors. Familial cancer aggregation may be explained by both genetic predisposition and by the fact that the members of a family tend to share the same habits, such as tobacco smoking, alcohol drinking, diet and occupation.^77^ During the past decade, several studies have explored the influence of genetic polymorphisms on the risk of laryngeal cancer. Most of these studies have been related to polymorphisms of genes implicated in the carcinogen-metabolizing enzymes, such as the enzyme genotypes of cytochrome P-450 (CYP) and glutathione-S-transferase (GST), which are the ones most frequently studied, while glucuronosyltransferase (UGT) and alcohol dehydrogenase (ADH) have also been examined. Polymorphisms of genes involved in DNA repair systems have also been studied.^78^ Some studies on carcinogen-metabolizing enzymes have found an elevated risk of laryngeal cancer for some polymorphisms, such as in those with the enzyme genotypes GSTT1-null,^79^ combined GSTM3 (AB or BB) and GSTM1-null,^80^ CYP1A1 (MspI MH or NcoI HT)^81^ and UGT1A10 codon 139 polymorphism.^82^ However, other studies have not found risks of laryngeal cancer for genetic polymorphisms in ADH1B, ADH1C, GSTM1 and GSTT1 after adjusting for tobacco smoking and alcohol consumption,^83^ or found a risk only when the GSTM1 and GSTT1 null genotypes were jointly present, but not for CYP1A1 and CYP2D6.^84^

Much more study will certainly be needed for the role of genetic polymorphisms linked to carcinogen-metabolizing enzymes to be clearly understood. There is a promising future for epidemiological studies on genetic polymorphisms, not only for laryngeal cancer, but also for cancer in general, lying in the exploration of the interaction between genetic polymorphisms and environmental factors.

## CONCLUSIONS

It remains clear that, even with the new evidence on a wide range of risk factors for laryngeal cancer, primary prevention for the majority of these tumors must be addressed through control of tobacco smoking and reduction in the consumption of alcoholic beverages. Patients without a history of tobacco smoking and use of alcohol who develop laryngeal cancer showed different characteristics from those of smokers or drinkers. Such individuals are on average 10 years older, show no male predominance and their lesions are mainly located in the glottis, which allows for early diagnosis and consequently higher survival rates.^85^

Laryngeal cancer incidence rates in the city of São Paulo, for the longest period that can be obtained for cancer incidence rates in Brazil (1969-1998), have declined since the late 1980s among males, but have been stable among females (Figure 1). This fact probably reflects lifestyle changes among women. The stable trend towards laryngeal cancer detected among women could worsen in the near future, given the various indications that women in younger age groups show higher tobacco smoking prevalence than for men in the same age groups.^86-89^

Nonetheless, independent of these trends, the high laryngeal cancer incidence patterns in Brazil, particularly in the southeastern and southern regions, require public health policies for the control of tobacco smoking and reduction of alcohol consumption, as well as reductions in carcinogen exposure in occupational settings.
